# Resources and Relationships in Disasters: Differences Among Small and Large Assisted Living Communities

**DOI:** 10.1093/geroni/igab046.1101

**Published:** 2021-12-17

**Authors:** Lindsay Peterson, Debra Dobbs, Joseph June, David Dosa, Kathryn Hyer

**Affiliations:** 1 University of South Florida, Bradenton, Florida, United States; 2 University of South Florida, School of Aging Studies, University of South Florida, Florida, United States; 3 University of South Florida, Tampa, Florida, United States; 4 Brown University, Providence, Rhode Island, United States; 5 University of South Florida, University of South Florida, Florida, United States

## Abstract

Disaster preparedness among assisted living communities (ALCs) has not been widely researched, despite the growth of ALCs and evidence of disability in this population. An additional issue of concern is the way in which ALCs vary, including variation by size. The purpose of this paper was to explore the experiences of ALCs in Florida that experienced Hurricane Irma in 2017 and how experiences varied by ALC size. Qualitative interviews and focus groups were conducted with representatives of small ALCs (<25 beds; n=32) and large ALCs (25+; n=38). Transcripts were analyzed using Atlas.ti version 8, and research team members collaborated to reach consensus on codes and further analyze differences based on ALC size. Results suggest there are differences among ALCs in their disaster preparedness and response, and these differences are related to size (e.g., access to resources, organizational characteristics). Implications for ALC resident wellbeing and future disaster planning will be discussed.

